# Supplementary material for: Content-rich biological network constructed by mining PubMed abstracts
Source: BMC Bioinformatics. 2004 Oct 8;5:147. doi: 10.1186/1471-2105-5-147 (PMC528731; doi:10.1186/1471-2105-5-147)
Supplement: Additional File 2 — The original results of the above study (non-essential files are deleted to keep the file size under the limit set by BMC bioinformatics). [file 1471-2105-5-147-S2.bz2 › chilibotAdditionalFile2/dip30/587ID9546397E10956/html/HFE_B2M.html]

 


 **HFE** and **B2M** 
  
Found 31 abstracts in PubMed, retrieved 30.  
 

 What does Google say? 
 PDF only 
| .edu only 

---

**Interactive relationship** (e.g. stimulation, inhibition, etc)

**Neutral relationship**- HLA H  [ **HFE** ]  associates with beta 2 microglobulin  [ **B2M** ] .  Ref: 9234244 Mol Med, 1997
- Defective iron homeostasis in beta 2 microglobulin  [ **B2M** ]  knockout mice recapitulates hereditary hemochromatosis  [ **HFE** ]  in man.  Ref: 8920884 J Exp Med, 1996
- **HFE**  is a nonclassical class I molecule that associates with beta 2 microglobulin  [ **B2M** ]  beta 2m and with the transferrin receptor.  Ref: 12456502 Blood, 2003
- In 90 per cent of cases a mutation is found in an MHC class like gene designated  **HFE** , involving a substitution at position 282 of the  **HFE**  protein and resulting in defective binding of beta 2 microglobulin  [ **B2M** ] .  Ref: 9725962 Lakartidningen, 1998
- Most patients are homozygous for a C282Y point mutation that abrogates the interaction of  **HFE**  with beta 2 microglobulin  [ **B2M** ]  beta 2 M and, thus, impairs its proper processing and expression on the cell surface.  Ref: 12464008 Biochem J, 2003
- The presence of beta 2 microglobulin  [ **B2M** ]  at the apical plasma membrane corresponds to the recently observed association of beta 2 microglobulin  [ **B2M** ]  with  **HFE**  and FcRn.  Ref: 11935295 Histochem Cell Biol, 2002
- The link between iron overload and  **HFE**  mutation is explained by the interaction between  **HFE**  protein, beta 2 microglobulin  [ **B2M** ]  and transferrin receptor, which is abolished by the C282Y mutation, but is not yet fully understood.  Ref: 10989541 Bull Acad Natl Med, 2000

**Non-interactive relationship** (e.g. studied together, co-existance, homology, etc.)

- The non covalent association of beta 2 microglobulin  [ **B2M** ]  with MHC class I molecules and MHC class I type molecules such as FcRn or the hemochromatosis  [ **HFE** ]  protein  **HFE**  is of major importance for their function, i.e., antigen presentation, IgG transport, and regulation of iron uptake, respectively.  Ref: 11935295 Histochem Cell Biol, 2002
- The function of  **HFE**  protein is unknown, but it is hypothesized that it acts in association with beta 2 microglobulin  [ **B2M** ]  and transferrin receptor 1 to regulate iron uptake from plasma transferrin by the duodenum, the proposed mechanism by which body iron levels are sensed.  Ref: 11943867 Proc Natl Acad Sci U S A, 2002
- The mutation abolishes the association of the  **HFE**  protein with beta 2 microglobulin  [ **B2M** ]  beta 2M, making the complex unable to gain the cell surface.  Ref: 9658732 Haematologica, 1998
- By co immunoprecipitation and Western blotting,  **HFE**  GFP protein formed a complex with endogenous transferrin receptor and beta 2 microglobulin  [ **B2M** ] , suggesting that this fusion protein has the function of  **HFE**  reported previously.  Ref: 10771090 Biochim Biophys Acta, 2000
- The following candidate genes have been examined in detail to determine whether polymorphisms in them may be responsible for this variation transferrin, transferrin receptor 1, transferrin receptor 2, ferritin L, ferritin H, IRP1, IRP2,  **HFE** , beta 2 microglobulin  [ **B2M** ] , mobilferrin calreticulin, ceruloplasmin, ferroportin, NRAMP1, NRAMP2 DMT1, haptoglobin, heme oxygenase 1, heme oxygenase 2, hepcidin, USF2, ZIRTL, duodenal cytochrome b ferric reductase DCYTB, TNFalpha, keratin 8, and keratin 18.  Ref: 12547238 Blood Cells Mol Dis< MedlineTA>Blood Cells Mol Dis, 2000
- The haemochromatosis  [ **HFE** ]  protein  **HFE**  induces an apparent iron deficient phenotype in H1299 cells that is not corrected by co expression of beta 2 microglobulin  [ **B2M** ] .  Ref: 12464008 Biochem J, 2003
- Functional data suggest that the mutation C282Y abolishes the association of the  **HFE**  protein with beta 2 microglobulin  [ **B2M** ]  beta 2M, making the complex unable to reach the cell surface.  Ref: 11941597 Zhonghua Yi Xue Yi Chuan Xue Za Zhi, 2002
- The first important step toward establishing the role of  **HFE**  in the pathogenesis of HC came with the recent observation that the C282Y mutation disrupts the binding of beta 2 microglobulin  [ **B2M** ]  to the  **HFE**  protein and as a result the mutant molecule is not expressed on the cell surface.  Ref: 9723005 Bioessays, 1998
- The expression and subcellular localization of DMT1 protein in 3 mouse models of iron overload were examined hypotransferrinemic Trf hpx mice,  **Hfe**  knockout mice, and  **B2m**  knockout mice.  Ref: 11159549 Blood, 2001
- The number of chromosomes at risk studied were 18 for HLA H  [ **HFE** ] , 14 for beta 2 microglobulin  [ **B2M** ]  and 10 for calreticulin.  Ref: 9234244 Mol Med, 1997
- Mice deficient in the product of the beta 2 microglobulin  [ **B2M** ]  beta 2 M class I light chain fail to express  **HFE**  and other MHC class I family proteins, and they have been found to manifest many characteristics of the HH phenotype.  Ref: 11309500 Proc Natl Acad Sci U S A, 2001
- The pathophysiological role of  **HFE**  is unclear it is expressed as a surface molecule on many cells and the C282Y mutation disrupts interactions with beta 2 microglobulin  [ **B2M** ] , thus preventing surface expression.  Ref: 9691000 Curr Opin Genet Dev, 1998
- Stable, recombinant, water soluble complexes of HLA A2 and HLA B27 were reconstituted from 125I labeled beta 2 microglobulin  [ **B2M** ]  beta 2m, a synthetic peptide, and HLA H  [ **HFE** ]  chain fragments expressed as inclusion bodies in the Escherichia coli cytoplasm.  Ref: 1517560 J Immunol, 1992
- MATERIALS AND METHODS We have sequenced the coding region and parts of introns of the HLA H  [ **HFE** ]  gene, the beta 2 microglobulin  [ **B2M** ]  gene, and the calreticulin mobilferrin gene of 1, and 5 hemochromatosis  [ **HFE** ]  patients, respectively, selecting those who were not homozygous for the 845A C282Y mutation.  Ref: 9234244 Mol Med, 1997
- We have moreover carried out a molecular analysis of candidate genes beta 2 microglobulin  [ **B2M** ] ,  **HFE** , and haem oxygenases 1 and 2 implicated in iron metabolism.  Ref: 11546828 J Med Genet, 2001
- To determine its role in hereditary hemochromatosis  [ **HFE** ]  HH, we used beta2 microglobulin knockout  **B2M**  mice that accumulate iron as in HH.  Ref: 12181184 Am J Physiol Gastrointest Liver Physiol, 2002
- The Cys282Tyr mutation disrupts a disulfid bond and thus abrogates binding of the mutant  **HFE**  protein to beta 2 microglobulin  [ **B2M** ]  and its presentation on the cell surface.  Ref: 10666843 Z Gastroenterol, 1999
- Compound mutant mice lacking both  **Hfe**  and its interacting protein, beta 2 microglobulin  [ **B2M** ]   **B2m** , deposit more tissue iron than mice lacking  **Hfe**  only, suggesting that another  **B2m**  interacting protein may be involved in iron regulation.  Ref: 10791995 J Clin Invest, 2000
- beta 2 Microglobulin  [ **B2M** ]  and  **HFE**  previously termed human leucocyte antigen H may be involved in stabilizing metal mobilferrin integrin to form this latter complex.  Ref: 10343352 Proc Nutr Soc, 1999
